# Incidence and Risk Factors of Diabetic Foot Ulcer: A Population-Based Diabetic Foot Cohort (ADFC Study)—Two-Year Follow-Up Study

**DOI:** 10.1155/2018/7631659

**Published:** 2018-03-15

**Authors:** Leila Yazdanpanah, Hajieh Shahbazian, Iraj Nazari, Hamid Reza Arti, Fatemeh Ahmadi, Seyed Ehsan Mohammadianinejad, Bahman Cheraghian, Saeed Hesam

**Affiliations:** ^1^Health Research Institute, Diabetes Research Center, Ahvaz Jundishapur University of Medical Sciences, Ahvaz, Iran; ^2^Department of Vascular Surgery, Ahvaz Jundishapur University of Medical Sciences, Ahvaz, Iran; ^3^Department of Orthopedic, Ahvaz Jundishapur University of Medical Sciences, Ahvaz, Iran; ^4^Infectious Disease Research Center, Ahvaz Jundishapur University of Medical Sciences, Ahvaz, Iran; ^5^Department of Neurology, Ahvaz Jundishapur University of Medical Sciences, Ahvaz, Iran; ^6^Department of Epidemiology and Biostatistics, School of Public Health, Ahvaz Jundishapur University of Medical Sciences, Ahvaz, Iran; ^7^Department of Epidemiology and Biostatistics, School of Public Health, Tehran University of Medical Sciences, Tehran, Iran

## Abstract

**Aim/Introduction:**

This study was carried out to assess the incidence and risk factors of diabetic foot ulcer (DFU).

**Materials and Methods:**

In this prospective cohort study in a university hospital, all the participants were examined and followed up for new DFU as final outcome for two years. To analyze the data, the variables were first evaluated with a univariate analysis. Then variables with *P* value < 0.2 were tested with a multivariate analysis, using backward-elimination multiple logistic regression.

**Results:**

Among 605 patients, 39 cases had DFU, so we followed up the remaining 566 patients without any present or history of DFU. A two-year cumulative incidence of diabetic foot ulcer was 5.62% (95% CI 3.89–8.02). After analysis, previous history of DFU or amputation [OR = 9.65, 95% CI (2.13–43.78), *P* value = 0.003], insulin usage [OR = 5.78, 95% CI (2.37–14.07), *P* value < 0.01], gender [OR = 3.23, 95% CI (1.33–7.83), *P* value = 0.01], distal neuropathy [OR = 3.37, 95% CI (1.40–8.09), *P* value = 0.007], and foot deformity [OR = 3.02, 95% CI (1.10–8.29), *P* value = 0.032] had a statistically significant relationship with DFU incidence.

**Conclusion:**

Our data showed that the average annual DFU incidence is about 2.8%. Independent risk factors of DFU development were previous history of DFU or amputation, insulin consumption, gender, distal neuropathy, and foot deformity. These findings provide support for a multifactorial etiology for DFU.

## 1. Introduction

Diabetes prevalence is increasing in developing and developed countries all over the world [1]. Diabetes complications are increasing too in this pandemic [2], making diabetes a major global health problem in different countries [3–5]. Among diabetes complications, managing diabetic foot remains as a major challenge for health care systems [6].

Diabetic foot is still the most frequent reason of hospitalization of patients with diabetes [7–10], and diabetes is the main cause of more than half of nontraumatic lower limb amputations [11–15]. In fact, every 30 seconds in the world, a lower limb is amputated due to diabetes [16], and it goes without saying that these amputations increase mortality rate [17].

About 15–25% of patients with diabetes may develop foot ulcer during their lifetime [15–20]. The annual risk of developing diabetic foot ulcer in patients with diabetes is estimated to be about 2%, but this risk in patients with previous history of foot ulceration is expected to increase to 17–60% over the next three years [21]. The prevalence of diabetic foot ulcer is reported to be 1.3–12% in different studies [19–22].

Since the development of foot ulcers and amputations are preventable and this condition can greatly affect the quality of life of patients [23], prevention of this complication can relieve direct and indirect cost burdens on society. Based on the results of a study conducted in Iran, of the total costs of diabetes complications in this country, 10.7% were related to diabetic foot (107.1 billion USD) [3].

Large cohort studies on diabetic foot ulcer (DFU) incidence are rare [16–24]. In Iran, there has not been any cohort study conducted on this complication; furthermore, the socio-economic differences between different societies can affect the incidence rates. Therefore, this cohort was designed to identify diabetic foot incidence and risk factors to help health providers to reduce the burden of this complication.

## 2. Materials and Methods

This population-based prospective cohort study named ADFC (Ahvaz Diabetic Foot Cohort), was done in a tertiary care diabetes clinic in Golestan Hospital, a university hospital in Ahvaz (south-west of Iran) with a diabetic foot clinic that is the first diabetic foot clinic in the province. This clinic was the main center of this cohort study. All patients with diabetes (with or without foot problems) were recruited from July 2014 and followed up until July 2016.

*Nonprobabilistic convenience sampling* was used to select the patients. The inclusion criteria were as follows: (1) age ≥ 18 years; (2) diagnosed with diabetes mellitus, both types 1 and 2; (3) able to complete the consent form; and (4) able to walk. The exclusion criteria were (1) severe disabling disease or inability to walk, (2) severe mental illness preventing informed consent, and (3) current foot ulcer.

The research followed the tenets of the Declaration of Helsinki and was also approved by the Ethics Committee of Ahvaz Jundishapur University of Medical Sciences. The procedure was described for all patients, and written informed consent was completed and signed by all of them at the first visit. A checklist including the following variables was completed for all participants: age, sex, blood pressure (BP), marital status, educational level, ethnicity, body mass index (BMI), waist circumference, job activity, smoking status, type of diabetes, diabetes duration, type of diabetes treatment (oral antidiabetes agents or insulin usage), diabetic retinopathy, diabetic nephropathy, having glucometer, history of DFU or amputation, present foot ulcer, preventive foot care, nail care, ill-fitting shoe, and patient training on feet.

Blood pressure was recorded as systolic and diastolic BP with a mercury sphygmomanometer. Marital status was categorized as single, married, dead partner, or divorced. Educational level was defined as illiterate, diploma or under diploma, and university degree. Ethnicities were defined as Arab, Fars, Lor, and other. BMI was measured in kg/m^2^. Waist circumference was considered as midline of the lower ribs and upper outer edge of the right iliac crest. Job activity was categorized to low, moderate, high, and no job. Smoking status was described as present smoker, former smoker, and no history of smoking. Diabetic retinopathy was considered if the patients' medical record included pupil dilation followed by examination by fundoscope (nonproliferative or proliferative retinopathy, clinically significant macular edema). Diabetic nephropathy was defined based on the patients' medical record report of 24 hours' urine collection test (microalbuminuria or overt proteinuria) or azotemia, dialysis, or kidney transplantation. Preventive foot care was considered as washing the feet and doing daily feet self-exam, drying the feet after washing, moisturizing the feet, not walking barefoot, not putting the feet close to the heater, and wearing slippers and suitable socks at home. Nail care was defined as not cutting toenails very short, not cutting the corners of the toenails, and filing the toenails. Ill-fitting footwear was described as slippers, tight shoe, or shoes with pressure points on the feet. Suitable socks were considered as cotton socks having a soft elastic band. Patient training on feet was defined as self-training (reading books or brochures, visiting websites, or watching films) or participation in scheduled individual or group sessions.

All participants were then examined. The examination included the following: skin and nails, types of foot deformity, neurologic foot exams, and vascular foot exams. DFU was defined as a full thickness skin defect at least Wagner stage 1 [25]. Pressure sensation examination was performed by 10-gram monofilaments (Owen Mumford, UK). Nylon monofilaments were applied perpendicular on four sites (1st, 3rd, and 5th metatarsal heads and plantar surface of distal hallux) of each foot. Areas of ulcer, calluses, necrotic tissues, and scars were avoided during the test. Loss of ability to detect the monofilament at even one site of examination was considered as distal neuropathy [26]. For vascular examination, dorsalis pedis, tibialis posterior, popliteal, and femoral pulses were assessed. ABI (ankle-brachial index) was measured by a handheld Doppler device (Huntleigh Diabetic Foot Kit, UK) and calculated by the following formula: ABI = (maximum systolic pressure of dorsalis pedis artery or tibialis posterior)/(maximum systolic pressure of brachial artery) separately for each leg. ABI = 0.9–1.3 was considered as normal, ABI = 0.4–0.9 as vascular disease, and ABI < 0.4 as severe vascular disease [27].

The questionnaires were completed, and the clinical exams were performed by a trained general physician.

A blood sample was obtained from each person to measure their HbA1c. It was tested in the Diabetes Research Center laboratory using the NycoCard technique to assess the plasma glucose control. HbA1c of less than 7% was considered as good glycemic control [28], 7-8% as relatively good control, and more than 8% as poor glycemic control.

Then all the patients were followed up for new DFU as final outcome for two years.

The data were recorded on SPSS version 20. To describe the variables, mean ± SD was used for continuous data, and frequency and percentage were used for categorical data. To analyze the data, the variables were first evaluated with a univariate analysis with diabetic foot ulcer incidence as variable. The statistical methods in this part were independent *t*-test (Mann–Whitney test if the data were not normally distributed), chi-square, and Fisher's exact test. Variables with *P* value < 0.2 were tested with a multivariate analysis, using backward-elimination multiple logistic regression. Thus, the most statistically significant variables were identified as risk factors. In this study, *P* value ≤ 0.05 was considered as significant.

## 3. Result

**Figure 1:**
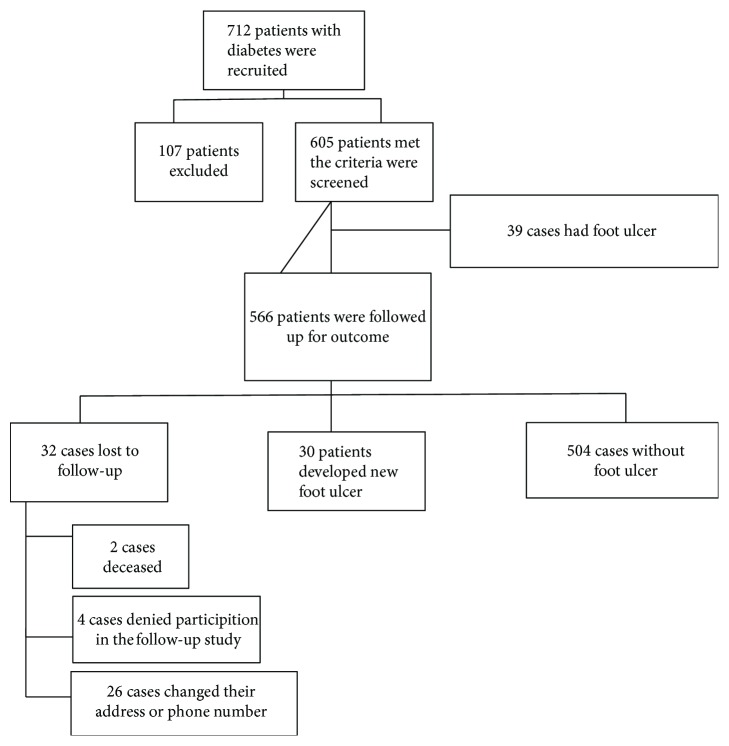
Diagram of the diabetic foot cohort participants.

Among all 712 patients with diabetes who were recruited in the study, 605 patients met the inclusion criteria. Thirty-nine cases (6.4%) had DFU, so we followed up 566 patients without any present or history of DFU for 24 months, of whom 32 were lost to follow-up (Figure 1). The mean duration of follow-up was 23.55 ± 2.39 months. The mean time at which the development of the ulcer occurred is 16.1 ± 6.6 months (minimum 4 months and maximum 24 months).

The mean (±SD) age of the participants was 53.52 ± 10.8 years. Of all cases, 307 (57.5%) were female and 521 (97.6%) had type 2 diabetes. The mean (±SD) duration of diabetes was 8.77 ± 6.87 years, and the mean HbA1c was 8.7 ± 1.7%. Seventy-nine cases (14.8%) had good glycemic control, 112 patients (21%) had HbA1c = 7-8%, and 343 cases (64.2%) had poor glycemic control.

The patients were followed up for diabetic foot ulceration as final outcome. The two-year cumulative incidence (risk) of diabetic foot ulcer was 5.62% (95% CI 3.89–8.02) (30 cases). The first-year incidence of foot ulceration was 1.50% (95% CI 0.70–3.05), and the second-year incidence was 4.18% (95% CI 2.70–6.36).

We excluded from data analysis patients who were lost to follow-up. Baseline characteristics of all participants and comparison between two groups (with and without diabetic foot ulcer) are shown in Table 1. In a univariate analysis, patients developing DFU were predominantly male, had longer duration of diabetes, had lower educational level, and were more likely to be smokers, compared with patients not developing DFU.

A univariate evaluation of risk factors of diabetic foot ulcer incidence and the comparison between patients developing and those not developing DFU are presented in Table 2. Patients developing DFU had more diabetic foot deformity, were less trained about their feet, and had more history of previous DFU or amputation, more decreased distal pulses, and more neuropathy, nephropathy, and retinopathy as opposed to patients not developing DFU.

In a multivariate logistic regression analysis, the variables with *P* value less than 0.2 were tested by backward elimination.  Table 3 describes the risk factors that were in the model. Finally, history of previous DFU or amputation, insulin usage, gender, distal neuropathy, and foot deformity had a statistically significant relationship with DFU incidence. Patient training on feet did not have any significant correlation with DFU incidence, but it was borderline significant [OR = 6.66, 95% CI (0.75, 59.19), *P* value = 0.089].

By controlling other variables, we found that previous history of DFU or amputation increased the odds of DFU by 9.65 times. The odds of DFU in patients using insulin were 5.78 times greater than those in patients using oral agent or just having lifestyle modification. The odds of DFU were 3.23 times more in men than in women. DFU was 3.51 times more likely in patients who had neuropathy than in patients without. Patients with foot deformity were 3.02 times more likely to develop DFU than were patients without (Table 3).

The comparison between the two groups in terms of preventive foot care is demonstrated in Table 4. Only 4 cases (0.7%) had a complete care of their feet.

## 4. Discussion

In this study, we sought to determine the incidence and risk factors of DFU for the first time in the south-west of Iran. In a prospective cohort, the two-year cumulative incidence of DFU was 5.62% (95% CI 3.89–8.02). The mean duration of follow-up was 23.55 ± 2.39 months. Baseline characteristics and probable risk factors were compared between patients developing and not developing DFU. Based on the multivariate analysis, independent risk factors of DFU development in this study were history of previous DFU or amputation, insulin usage, gender, distal neuropathy, and foot deformity.

Prospective estimates of DFU incidence are rare [1-29]. Few studies, like ours have reported outcome in patients without any active or past foot ulcer [1]. The average annual DFU incidence in the present study was about 2.8%, which is comparable to that of other studies [22–30]. These studies are prospective (except Ramsey et al. [[22]]) with older patients and nearly the same duration of diabetes in comparison with our study. Leese et al. study showed 4.7% DFU incidence during 1.7 years' follow-up [26]. Abbott et al. reported 2.2% average annual incidence of DFU [24]. Crawford et al.'s study reported 1.93% new foot ulcer during an average 1-year follow-up [30], while Ramsey et al.'s study reported DFU incidence to be 5.8% over 3 years of observation [22]. A prospective study by Hurley et al., involving 18 months' follow-up of 563 patients, reported the same incidence too, but their patients had lower duration of diabetes [11]. Jiang et al.'s study, a cohort in China with 678 patients, reported DFU incidence to be 8.1% in a 1-year follow-up, which was higher than that in our study and that of western countries according to their own report [9].

According to the literature, the risk factors of foot ulceration vary from one study to another, but some of them are common. First, we used a univariate analysis to demonstrate the simple relationships between DFU and its potential risk factors; however, many of these variables are substitutes for real factors. Consistent with other studies, previous history of DFU or amputation was the most associated risk factor of DFU in our study [1–32].

This is logical because patients with a history of ulceration may be predisposed to different micro- and macrovascular dysfunctions or peripheral neuropathy.

We found that patients treated with insulin were more likely to develop foot ulcer than were patients whose diabetes is managed with oral glycemic agent or lifestyle modification alone. This may be because of the fact that when patients acquiesce to start insulin, they may already have diabetes for a long time with greater associated complications. It could also be because of another confounding factor that we did not assess in our study, so insulin might play the role of that confounder. This might be due to the unequal number of patients in the two groups after the 2-year follow-up (those developing foot ulcer were 30 as opposed to 504 who did not develop foot ulcer). Nevertheless, this finding is compatible with few studies [9–33]. In a systematic review, 7 studies out of 16 reported an association between DFU and insulin treatment [1]. It seems that further studies are needed to elaborate on this variable by eliminating the possible confounding factors and providing more details.

The predominance of male patients developing DFU was another finding of this study, which was consistent with other studies [1–32]. This could be explained by the fact that men have more outside activity than have women, which may lead to more foot exposure to different risks and more plantar pressure on their feet. Some studies reported male association with DFU just in a univariate analysis, but it was not significant in a multivariate analysis [9–30].

Another risk factor we identified was distal neuropathy, which is a well-known risk factor for developing foot ulcers. Many studies recommend the use of 10 g monofilament, and this finding was similar to that of some other prospective studies [11–33] and cross-sectional studies [34].

Foot deformity was another risk factor of DFU in this study comparable with some studies [24–31]. In one study [33], it was significant just in a univariate analysis. Few patients in our study had foot deformity (9.4%) including hammer toe, hallux valgus, bunion, prominent metatarsal head, and just one Charcot joint.

Logically, we expected patient training on feet to have a relationship with DFU development, but in contrast to our previous study [10], it did not have any significant relationship with foot ulceration; however, it was borderline significant. This might be because of the severe unbalance between trained and untrained patients (Table 2).

Unrelated foot ulcer risk factors in a multivariate analysis were diabetes duration, educational level, marital status, job activity, smoking, glycemic control (HbA1c), retinopathy, nephropathy, and decreased peripheral pulses in this study. Some of these factors such as hyperglycemia, smoking, and decreased peripheral pulses have been described as risk factors in other studies [11, 14]. In this study, the small number of patients developing foot ulcer may have led to this result. Alternatively, smoking cessation in some patients or intensive blood glucose control when developing a complication may bring about these results.

Our study has a number of limitations. First, although this study was performed in a cohort design, our patients were from a university hospital, and this may affect the results by selection bias. However, our hospital was the referral center and the focal point of diabetes in the province, and this can be the strength of the study to have different patients with different conditions. Second, the small size of subgroups in analysis may lead to less precision in our estimates, and this was the reason why a wide range of confidence intervals for some estimates was obtained. Third, we did not consider some potential confounders in the occurrence of new foot ulceration such as health care provision level and patient behavioral factors like compliance with training on their foot care. Finally, differences in methods of neuropathy assessment may affect the results to be compared with those of other studies.

The strength of this study was its low lost to follow-up rate in comparison with that of other studies [11–30]. We have low missing data too. This study was the first population-based prospective cohort of diabetic foot in this area, and this paper is the first report of this study. Additionally, all patients with diabetes have to undergo an annual foot examination to identify risk factors of foot ulceration [28]. The results of this study could support this suggestion to reduce DFU incidence, but it is better to assess the cost-effectiveness of this annual screening in future studies. We recommend that future studies have a larger sample size and longer follow-up period.

In conclusion, our data reported the average annual DFU incidence to be about 2.8%. Independent risk factors of DFU development were history of previous DFU or amputation, insulin usage, gender, distal neuropathy, and foot deformity. This finding provides support for a multifactorial etiology of DFU.

## Figures and Tables

**Table 1:** Baseline characteristics of all participants and comparison between two groups (developing and not developing DFU).

Characteristics	All patients (*n* = 534)	Patients developing DFU (*n* = 30)	Patients not developing DFU (*n* = 504)	*P* value
*Age (years)* ^∗^	53.52 ± 10.81	55.9 ± 13.47	53.38 ± 10.63	0.214
*Gender*				
Female	307 (57.5)	9 (30)	298 (59.1)	**0.002**
Male	227 (42.5)	21 (70)	206 (40.9)	
*Diabetes duration (year)* ^∗^	8.77 ± 6.87	13.46 ± 8.48	8.49 ± 6.67	**0.001**
*BMI (kg/m^2^)* ^∗^	28.63 ± 4.42	28.33 ± 4.91	28.65 ± 4.40	0.700
*Waist circumference (cm)* ^∗^	95.22 ± 11.02	95.33 ± 12.46	95.22 ± 10.94	0.710
*Blood pressure (mmHg)* ^∗^				
Systolic BP	128.74 ± 11.87	129.67 ± 12.99	128.68 ± 11.81	0.879
Diastolic BP	81.11 ± 4.92	81.33 ± 5.71	81.10 ± 4.88	0.918
*Ethnicity*				0.974
Fars	158 (29.6)	8 (26.7)	150 (29.8)	
Arab	272 (50.9)	16 (53.3)	256 (50.8)	
Lor	86 (16.1)	5 (16.7)	81 (16.1)	
Other	18 (3.4)	1 (3.3)	17 (3.4)	
*Education*				**0.017**
Illiterate	129 (24.2)	11 (36.6)	118 (23.4)	
≤Diploma	366 (68.5)	14 (46.7)	352 (69.9)	
University degree	39 (7.3)	5 (16.7)	34 (6.7)	
*Marital status*				0.269
Single	12 (2.2)	0 (0)	12 (2.4)	
Married	498 (93.3)	27 (90)	471 (93.4)	
Divorced or dead partner	24 (4.5)	3 (10)	21 (4.2)	
*Job activity*				0.097
Low	446 (83.5)	21 (70)	425 (84.3)	
Moderate	41 (7.7)	5 (16.7)	36 (7.1)	
High	17 (3.2)	1 (3.3)	16 (3.2)	
No job	30 (5.6)	3 (10)	27 (5.4)	
*Smoking status*				**0.013**
Present	26 (4.9)	3 (10)	23 (4.6)	
Former	43 (8.1)	6 (20)	37 (7.3)	
No smoker	465 (87.1)	21 (70)	444 (88.1)	

DFU: diabetic foot ulcer; BP: blood pressure. ^∗^These variables are described as mean ± SD; other are presented as *N* (%).

**Table 2:** Univariate evaluation of risk factors of diabetic foot ulcer incidence and the comparison between patients developing and not developing DFU.

Variable	All patients (*n* = 534)	Patients developing DFU (*n* = 30)	Patients not developing DFU (*n* = 504)	*P* value
*Neuropathy*				**<0.001**
Yes	172 (32.2)	21 (70)	151 (30)	
No	362 (67.8)	9 (30)	353 (70)	
*Decreased distal pulses*				0.99
Yes	6 (1.1)	0 (0)	6 (1.1)	
No	528 (98.9)	30 (100)	498 (98.9)	
*ABI*				0.28
Normal	528 (98.9)	29 (96.7)	499 (99)	
Abnormal	6 (1.1)	1 (3.3)	5 (1)	
*Foot deformity*				**0.001**
Yes	50 (9.4)	8 (26.7)	42 (8.3)	
No	484 (90.6)	22 (73.3)	462 (91.7)	
HbA1c^∗^	8.73 ± 1.73	9.31 ± 1.79	8.69 ± 1.72	0.058
*Retinopathy*				
Yes	106 (19.9)	12 (40)	94 (18.7)	**0.004**
No	428 (80.1)	18 (60)	410 (81.3)	
*Nephropathy*				**0.004**
Yes	47 (8.8)	7 (23.3)	40 (7.9)	
No	487 (91.2)	23 (76.7)	464 (92.1)	
*History of previous DFU or amputation*				**<0.001**
Yes	11 (2.1)	6 (20)	5 (1)	
No	523 (97.9)	24 (80)	499 (99)	
*Insulin consumption*				**<0.001**
Yes	163 (30.5)	22 (73.7)	141 (28)	
No	371 (69.5)	8 (26.7)	363 (72)	
*Patient training on feet*				0.104
Yes	77 (14.4)	1 (3.3)	76 (15.1)	
No	457 (85.6)	29 (96.7)	428 (84.9)	
*Ill-fitting footwear*				0.433
Yes	338 (63.3)	21 (70)	317 (62.9)	
No	196 (36.7)	9 (30)	187 (37.1)	
*Preventive foot care*				0.999
Yes	4 (0.7)	0 (0)	4 (0.8)	
No	530 (99.3)	30 (100)	500 (99.2)	
*Toenail care*				0.246
Yes	32 (6)	0 (0)	31 (6.2)	
No	502 (94)	30 (100)	473 (93.8)	
*Visit the physician less than 3 months ago*				0.964
Yes	358 (67)	20 (66.7)	338 (67.1)	
No	176 (33)	10 (33.3)	166 (32.9)	
*Having glucometer*				0.840
Yes	383 (71.7)	22 (73.3)	361 (71.6)	
No	151 (28.3)	8 (26.7)	143 (28.4)	

∗ is presented in mean ± SD; other variables are presented as *N* (%).

**Table 3:** Independent risk factors of DFU using univariate and multivariate logistic regression analysis.

Risk factors (base)	Unadjusted OR	95% CI	*P* value	Adjusted OR	95% CI	*P* value
History of previous DFU or amputation (no)	24.95	7.11–87.57	<0.001	9.65	2.13–43.78	0.003
Insulin treatment (no)	7.08	3.08–16.27	<0.001	5.78	2.37–14.07	<0.01
Gender (female)	3.38	1.52–7.52	0.003	3.23	1.33–7.83	0.01
Distal neuropathy (no)	5.46	2.44–12.19	<0.001	3.37	1.40–8.09	0.007
Foot deformity (no)	4.00	1.68–9.54	0.002	3.02	1.10–8.29	0.032
Patient training on foot care (yes)	5.15	0.69–38.37	0.110	6.66	0.75–59.19	0.089

OR: odds ratio; CI: confidence interval.

**Table 4:** Comparison of preventive foot care in patients developing and not developing DFU.

Variable	All patients (*n* = 534)	Patients developing DFU (*n* = 30)	Patients not developing DFU (*n* = 504)	*P* value
*Washing the feet daily*				0.976
Yes	304 (56.9)^∗^	17 (56.7)	287 (56.9)	
No	230 (43.1)	13 (43.3)	217 (43.1)	
*Daily feet self-examination*				0.266
Yes	120 (22.5)	4 (13.3)	116 (23)	
No	414 (77.5)	26 (86.7)	388 (77)	
*Drying the feet after washing*				0.757
Yes	54 (10.1)	2 (6.7)	52 (10.3)	
No	480 (89.9)	28 (93.3)	452 (89.7)	
*Moisturizing the feet*				0.617
Yes	108 (20.2)	5 (16.7)	103 (20.4)	
No	426 (79.8)	25 (83.3)	401 (79.6)	
*Walking barefoot*				0.999
Yes	14 (2.6)	0 (0)	14 (2.8)	
No	520 (97.4)	30 (100)	490 (97.2)	
*Putting the feet close to the heater*				0.561
Yes	60 (11.2)	2 (6.7)	58 (11.5)	
No	474 (88.8)	28 (93.3)	446 (88.5)	
*Wearing slippers at home*				0.222
Yes	39 (7.3)	0 (0)	39 (7.7)	
No	495 (92.7)	30 (100)	465 (92.3)	
*Wearing suitable socks*				0.999
Yes	48 (9)	2 (6.7)	46 (9.1)	
No	486 (91)	28 (93.3)	458 (90.9)	

^∗^All are presented in number (%).
